# The impact of different ale brewer’s yeast strains on the proteome of immature beer

**DOI:** 10.1186/1471-2180-13-215

**Published:** 2013-09-30

**Authors:** Torben Sune Berner, Susanne Jacobsen, Nils Arneborg

**Affiliations:** 1 Food Microbiology, Department of Food Science, University of Copenhagen, DK-1958, Frederiksberg, Denmark; 2 Enzyme and Protein Chemistry, Department of Systems Biology, Technical University of Denmark, Building 224, DK-2800, Kgs. Lyngby, Denmark

**Keywords:** 2-DE, Beer, Fermentation, Brewer’s yeast, Secreted proteins

## Abstract

**Background:**

It is well known that brewer’s yeast affects the taste and aroma of beer. However, the influence of brewer’s yeast on the protein composition of beer is currently unknown. In this study, changes of the proteome of immature beer, i.e. beer that has not been matured after fermentation, by ale brewer’s yeast strains with different abilities to degrade fermentable sugars were investigated.

**Results:**

Beers were fermented from standard hopped wort (13° Plato) using two ale brewer’s yeast (*Saccharomyces cerevisiae*) strains with different attenuation degrees. Both immature beers had the same alcohol and protein concentrations. Immature beer and unfermented wort proteins were analysed by 2-DE and compared in order to determine protein changes arising from fermentation. Distinct protein spots in the beer and wort proteomes were identified using Matrix-assisted laser desorption-ionization time-of-flight mass spectrometry (MALDI-TOF-MS) and MS/MS and revealed common beer proteins, such as lipid transfer proteins (LTP1 and LTP2), protein Z and amylase-protease inhibitors. During fermentation, two protein spots, corresponding to LTP2, disappeared, while three protein spots were exclusively found in beer. These three proteins, all derived from yeast, were identified as cell wall associated proteins, that is Exg1 (an exo-β-1,3-glucanase), Bgl2 (an endo-β-1,2-glucanase), and Uth1 (a cell wall biogenesis protein).

**Conclusion:**

Yeast strain dependent changes in the immature beer proteome were identified, i.e. Bgl2 was present in beer brewed with KVL011, while lacking in WLP001 beer.

## Background

Head foam stability and haze absents (clarity) are the main characteristics associated with fresh and pleasant beer [1]. Proteins in beer have an effect on both haze formation and foam stability, as polypeptides of storage proteins from barley aggregate and form haze during maturation of beer while other proteins form complexes with hop acids that stabilize the beer foam [2,3]. In recent years, focus on proteomic analysis of beer has become a way to unravel how beer proteins evolve during the production process of beer and how proteins in beer interact. The most comprehensive proteome studies report that beer proteomes consist of only 20–30 different proteins from barley [4-6], all heat stable and protease resistant [7]. However, it is not only proteins from barley that are identified in the beer proteome; also proteins from yeast and maize have been identified [4,5,8,9]. The two most predominant, barley-derived proteins in beer are lipid transfer protein 1 (LTP1) and protein Z, estimated to contribute for more than 25% of the total amount of proteins in beer [9,10]. Different inhibitors involved in the pathogenic defence of barley are found in the final beer, such as α-amylase inhibitor (BDAI-I), trypsin/α-amylase inhibitor (pUP13) and trypsin inhibitors (CMe, CMa, CMb) [11,12]. Perrocheau *et al*. (2005) showed that hordeins, i.e. storage proteins from barley, and many of the trypsin/α-amylase inhibitors from barley, vanish during the process of making beer (wort boiling and fermentation) and only half of the proteins identified in barley grain were also present in beer. Other studies used two-dimensional gel electrophoresis (2-DE) to discover proteins involved in head foam and beer haze formation [13-16] and the influence of malt modification and processing [6,14]. Proteins derived from brewer’s yeast have also been identified in beer, although the range of identified proteins vary from 2–4 proteins [8,17] to 31 proteins [5] and 40 protein fragments [4]. The origin of the identified proteins also vary from proteins localized in the cytosol, such as enolase and triosephosphate isomerase, to proteins like Swc4 and Uth1 that are associated to the cell wall [4,5,8]. One common feature for all beer proteome studies, so far, is that commercial beers have been used where no information on raw materials, choice of brewer’s yeast strain, or fermentation conditions have been given.

In this study, we used two ale brewer’s yeast strains, differing in their ability to consume fermentable sugars, for brewing beer under controlled conditions to determine the protein changes caused by fermentation, and to explore if there are any yeast strain dependent changes of the beer proteome.

## Methods

### Yeast strains and media

The yeast strains (WLP001 and KVL011) used in this study were ale brewer’s yeast strains, belonging to the species *Saccharomyces cerevisiae*, obtained from White Labs (WL, San Diego, California, USA) and our own collection (KVL) at the Department of Food Science, Food Microbiology, University of Copenhagen, respectively. Yeast strains were grown in 0.3% malt extract, 0.3% yeast extract, 0.5% peptone, 1% glucose, pH5.6 (MYGP) or in standard hopped wort (13° Plato) from Skands Brewery (Skands, Brøndby, Denmark).

### Beer fermentation

Aerobic propagation of yeast was started from a single colony on a MYGP-agar plate in 10 ml MYGP, in duplicate. After incubation at 20°C for 24 h, the yeast suspensions were transferred to 100 ml MYGP in 250 ml Erlenmeyer flasks with aeration at 200 rpm. Yeast suspensions were transferred after two days at 20°C to 400 ml double concentrated MYGP and incubated for 24 h at 20°C. Yeast cells were harvested (3000 g, 10 min, 20°C) and inoculated at 7 × 10^6^ cells/ml in 2 litres of wort saturated with air. Fermentations were carried out in biological duplicates in 2.5-liters European Brewing Convention (EBC) tubes at 18°C for 155 hours. To monitor the fermentation, samples of culture broth were collected aseptically twice on a daily basis from the top of the EBC-tubes for 155 hours. Yeast growth was followed by measuring the optical density at 600 nm (OD_600_)(UV-1800; Shimadzu Scientific Instruments) and pH (pHM220; Radiometer Analytical SAS).

### Sugar and ethanol determination

Samples were filtrated using a 0.22 μm sterile filter and kept at -20°C until analysis. Sugar and ethanol concentrations were determined using a HPLC (HP series 1100, Hewlett-Packard Company, USA) with a MicroGuard cation H cartridge followed by an Aminex HPX-87H column (Bio-Rad Laboratories, Hercules, USA) connected to a RI detector (HP1047A, Hewlett-Packard Company, USA). The column was eluted with a degassed mobile phase containing 2.5 mM H_2_SO_4_, pH 2.75, at 50°C and at a flow rate of 0.6 ml/min.

### Beer protein sample preparation

Samples of beer proteins were collected aseptically from the top of the fermentation vessel at the end of fermentation (after 155 hours). The culture broth samples were filter sterilized using a 0.22 μm filter to remove yeast cells and degas the sample. Salts and free amino acids were removed on a Sephadex G25 desalting column (PD 10, GE Life Sciences) using 20% Mcllvaine buffer (0.2 M Na_2_HPO_4_, 0.1 M citric acid) pH 4.4 added 5% ethanol in all steps. After desalting, proteins were concentrated by lyophilisation and dissolved in 8 M urea, 2 M thiourea and 3% 3-[(3-cholamidopropyl) dimethylammonio]-1-propanesulfonate (CHAPS). Protein concentrations were determined using the 2D Quant kit (GE Life Sciences) according to the manufacturer’s protocol, with bovine serum albumin as a standard.

### Two-dimensional gel electrophoresis (2-DE)

2-DE was run according to Jacobsen *et al.* (2011) [18] with minor modifications. Prior to 2-DE, rehydration buffer (8 M urea, 3%w/v CHAPS, 1%v/v IPG buffer, pH 3–10 [GE Life Sciences], 100 mM dithiothreitol [DTT), 1%v/v DeStreak Reagent [GE Life Sciences]) was added to samples of beer proteins (corresponding to 600 μg protein) to a final volume of 350 μl. Samples were centrifuged (14,000 g, 3 min) and applied to an IPG strip (18 cm, linear pH gradient 3–10, GE Healthcare). Isoelectric focusing (IEF) was run on an Ettan IPGphor (GE Life Sciences) for a total of 75.000 Vh as described in [19]. After IEF, IPG strips were reduced for 20 min by 10 mg/ml DTT in equilibration buffer (50 mM Tris–HCl, pH 8.8, 6 M urea, 30% [v/v] glycerol, 2% [w/v] sodium dodecyl sulfate (SDS) and 0.01% [w/v] bromophenol blue) followed by alkylation for 20 min with 25 mg/ml iodoacetamide in equilibration buffer [18]. Electrophoresis in the second dimension was carried out using 12.5% acrylamide gels (3% C/0.375% bisacrylamide) and was run on an Ettan^TM^ DALT *six* Electrophoresis Unit (GE Life Sciences) according to the manufacturer’s protocol. Proteins were stained by Blue Silver stain over night and de-stained in water until background was negligible [20]. Each biological replicate was done in technical triplicates to ensure reproducibility.

### In-gel trypsinolysis and MALDI-TOF-MS

Protein spots were manually excised from the Blue Silver stained 2D-gels and subjected to in-gel tryptic digestion according to [21], omitting the reduction and alkylation steps as this was done prior to 2-DE. Briefly, protein spots were de-stained in 40% ethanol, dehydrated in 100% acetonitrile, rehydrated in 10 mM NH_4_HCO_3_ with 12.5 ng ng/μl trypsin (Promega, porcine sequencing grade), incubated on ice for 45 min, and finally diluted five fold with 10 mM NH_4_HCO_3_ and incubated at 37°C over night. Supernatant was removed from the gel and stored at -20°C until analysis.

Samples were added on an Anchorchip™ (Bruker-Daltonics, Bremen, Germany) as described by [21]. Mass determinations were determined by an Ultraflex II MALDI-TOF mass spectrometer (Bruker-Daltonics, Bremen, Germany) in positive reflector mode for peptide mass mapping or peptide fragment ion mapping. Spectra were externally calibrated using a tryptic digest of β-lactoglobulin. The obtained spectra were analysed using Flex-Analysis 3.0.96 and Biotools 3.1 software program before searching an in-house MASCOT server (http://www.matrixscience.com) against the genomes of *Saccharomyces cerevisiae* and *Hordeum vulgare*. The following parameters were used for protein identification: allowed global modification; carbamidomethyl cysteine; variable modification; oxidation of methionine; missed cleavages - 1; peptide tolerance – 80 ppm and MS/MS tolerance ± 0.5 Da. Trypsin autolysis products were used for internal mass calibration. Proteins were positively identified, when a significant MASCOT score and at least three matched peptides in MS analysis, or one matched peptide in MS/MS analysis (Additional file 1), occurred.

### Statistical analysis

Beer properties are represented as the mean values ± standard error of the mean (SEM) from two biological replicates with at least duplicate measurements. Statistical analysis was performed by a two tailed T-test using StatPlus software (AnalystSoft, Inc.). Probabilities less than 0.05 were considered significant.

## Results

### Beer fermentation

To investigate the influences of fermentation and brewer’s yeast on the beer proteome, we used two different ale brewing yeast strains (WLP001 and KVL011) to produce beer. The yeast strains were chosen based on their different attenuation degrees; i.e. their different abilities to deplete fermentable sugars. The strain KVL011, which is an industrial ale brewer’s yeast strain, is reported to have an attenuation degree of 85%, while the WLP001, which is a micro brewer’s yeast strain, is reported to attenuate 73–80% (whitelabs.com).

**Figure 1:**
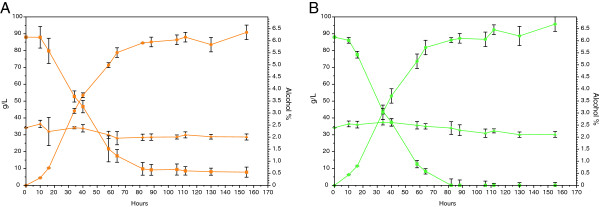
**Fermentation profiles for ale brewer’s yeast strains WLP001 (A) and KVL011 (B) grown in 2 L standard hopped wort.** Fermentable sugars (■) and dextrins (▲) are shown in g/l, and ethanol (●) is shown in % (v/v). Values are means for two biological replicate fermentations and error bars indicate standard error of the mean (SEM).

**Figure 2:**
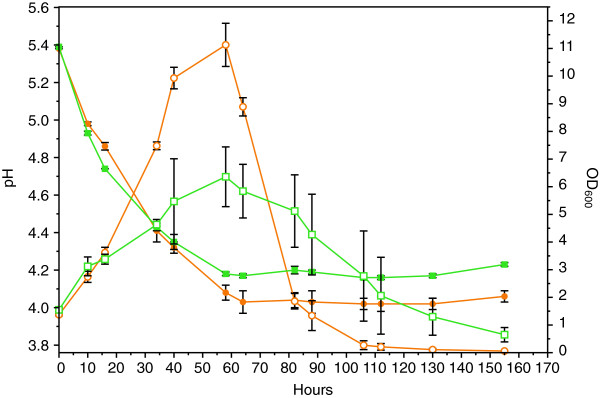
**Acidification and cell division during 2 L beer fermentations with ale brewer’s yeast strains WLP001 (●) and KVL011 (■).** pH is represented with filled symbols and OD_600_ with open symbols. Values are means for two biological replicate fermentations and error bars indicate standard error of the mean (SEM).

The two beers were brewed using standard hopped wort (13° Plato) in EBC tubes. As expected, some fermentable sugars were still present in the beer brewed with WLP001, while all fermentable sugars were depleted by the KVL011 yeast strain (Figure 1, Table 1). In both beers, the yeast cells were growing for 60 hours, reaching OD_600_ values of 11.3 ± 0.8 and 6.4 ± 1.1 for WLP001 and KVL011, respectively, before onset of flocculation (Figure 2). The flocculation ability of WLP001 was higher than for KVL011, as ten fold less yeast cells were in suspension for the beer brewed with yeast strain WLP001 after 130 hours compared to the beer brewed with KVL011 (Figure 2).

**Table 1:** Properties of brewed beers and wort

**Beer**	**Sugar content (g/l)**		**Protein concentration (mg/ml)**	**Ethanol % (v/v)**
	**Fermentable**	**Dextrins**		
WPL001	7.8 ± 3.0	28.7 ±1.8	0.42 ± 0.01	6.4 ± 0.2
KVL011	0.0 ± 0	30.2 ±1.7	0.29 ± 0.05	6.7 ± 0.3
Wort	88.0 ± 2.2	34.21 ± 1.9	0.49 ± 0.01	0.0 ± 0

For both yeast strains, the pH dropped from 5.5 to 4.1 (Figure 2) and the ethanol concentration increased from 0 to 6.4-6.7% (v/v) (Figure 1, Table 1) after 60 hours of fermentation. Furthermore, a decrease in the protein concentration was observed during fermentation. In the beginning of the fermentation, the wort contained 0.50 mg/ml, while in the final beer the protein concentration was 0.42 and 0.29 mg/ml for beers brewed with yeast strain WLP001 and KVL011, respectively (Table 1).

The ethanol and protein concentrations between the two beers were not significantly different (Figure 1, Table 1).

### Protein identification

**Figure 3:**
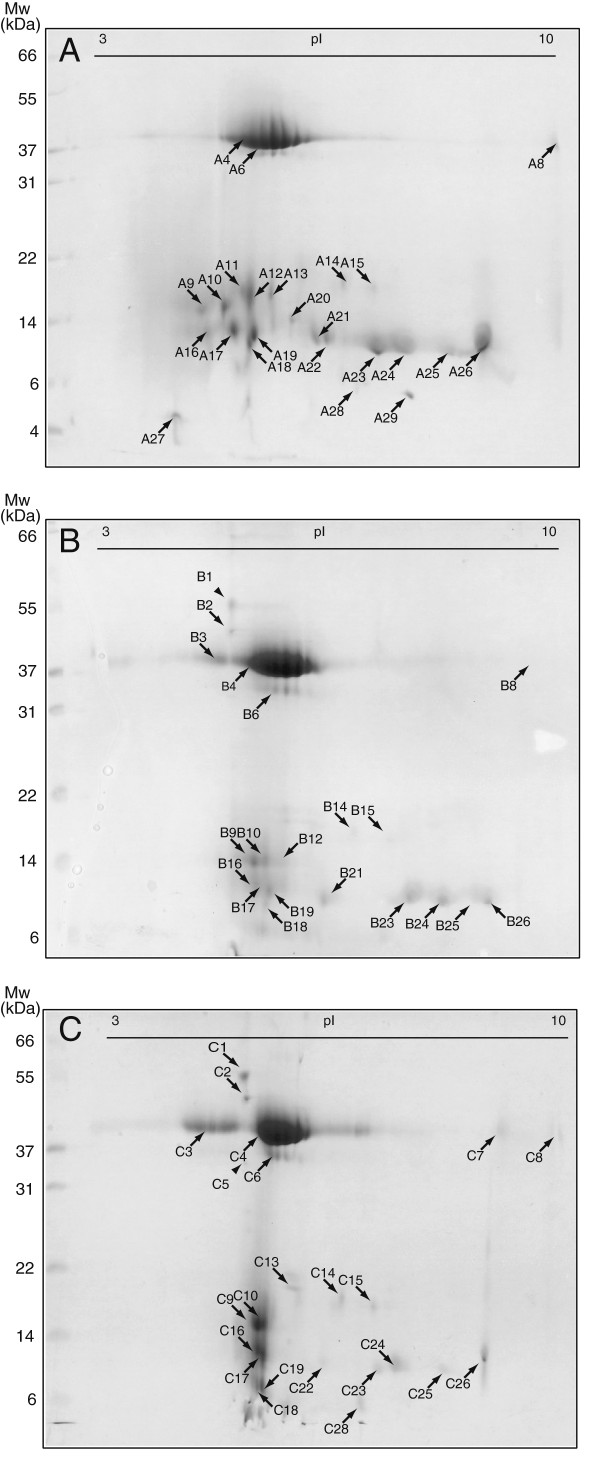
**2-DE gel protein profiles of wort (A) and beer fermented with WLP001 (B) or KVL011 (C).** Black and two arrow heads (B1 and C5) indicate protein spots subjected to MALDI-TOF-MS and MS/MS analysis, respectively.

Proteins from the unfermented wort and the two beers were separated by 2-DE to estimate differences in protein composition, caused by different yeast strains during the fermentation process with the unfermented wort as a reference (Figure 3). All distinct protein spots from each proteome were analysed by MALDI-TOF-MS or MS/MS. From the 90 distinct protein spots picked, we identified 66 spots that originated from 10 unique proteins. The most dominant proteins found in wort and beer were identified as protein Z, LTP1 and the barley-derived inhibitors pUP13, CMe, CMa and BDAI-I (Figure 3, Table 2). LTP1 was identified in four discrete protein spots with a pI ranging from 6.3 to 9.1 in wort (Figure 3; spot A22, A24, A25, A26), as compared to five locations in the WLP001 and KVL011 beers (Figure 3; spot B21, B23, B24, B25, B26, C22, C23, C24, C25, C26). A fragment of the barley storage protein D-hordein was only detected in wort (Figure 3; spot A18, Table 2).

**Table 2:** List of beer proteins identified by MALDI-TOF-MS and MS/MS

			**Theoretical values**					
**Spot ID**	**Protein name**	**Accession no.**	**M**_**r**_**(Da)**	**pI**	**Score**^**a**^	**Sequence coverage (%)**	**No. of peptide**	**MS/MS (sequnece of matched peptides)**^**b**^
A6	Protein Z-type serpin	gi|1310677	43307	5.61	110	32	11	
A8	Protein Z-type serpin	gi|1310677	43307	5.61	156	22	12	
A9	Trypsin/amylase inhibitor pUP13	gi|225102	15370	5.35	107	29	7	
A10	Trypsin/amylase inhibitor pUP13	gi|225102	15370	5.35	127	38	10	
A12	Trypsin/amylase inhibitor pUP13	gi|225102	15370	5.35	86	58	9	
A16	Alpha-amylase inhibitor BDAI-I	gi|123970	14045	5.36	100	53	8	
A17	Alpha-amylase inhibitor BDAI-I	gi|123970	14045	5.36	98	53	8	
A18	D-hordein	gi|671537	51154	7.60	207	9	6	
A19	Alpha-amylase inhibitor BDAI-I	gi|123970	14045	5.36	91	33	8	
A22	Lipid transfer protein 1	gi|19039	10145	8.91	243	21	4	
A24	Lipid transfer protein 1	gi|19039	10145	8.91	296	68	5	
A25	Lipid transfer protein 1	gi|19039	10145	8.91	100	68	6	
A26	Lipid transfer protein 1	gi|19039	10145	8.91	128	93	7	
A28	Lipid transfer protein 2	gi|128377	10806	6.78	77	37	4	
A29	Lipid transfer protein 2	gi|128377	10806	6.78	72	37	4	
B1	Uth1	gi|486485	47576	4.45	90	4	1	K.TQWPSEQPSDGR.S
B2	Exg1	gi|37926403	47335	4.45	257	23	9	
B3	Protein Z-type serpin	gi|1310677	43307	5.61	178	27	9	
B4	Protein Z-type serpin	gi|1310677	43307	5.61	118	33	11	
B6	Protein Z-type serpin	gi|1310677	43307	5.61	178	27	9	
B8	Protein Z-type serpin	gi|1310677	43307	5.61	120	26	10	
B9	Trypsin/amylase inhibitor pUP13	gi|225102	15370	5.35	110	54	8	
B10	Trypsin/amylase inhibitor pUP13	gi|225102	15370	5.35	98	52	7	
B12	Trypsin/amylase inhibitor pUP13	gi|225102	15370	5.35	109	55	9	
B16	Alpha-amylase inhibitor BDAI-I	gi|123970	14045	5.36	115	29	5	
B17	Alpha-amylase inhibitor BDAI-I	gi|123970	14045	5.36	94	53	8	
B19	Alpha-amylase inhibitor BDAI-I	gi|123970	14045	5.36	99	15	3	
B21	Lipid transfer protein 1	gi|19039	10145	8.91	252	52	6	
B23	Lipid transfer protein 1	gi|19039	10145	8.91	595	74	8	
B24	Lipid transfer protein 1	gi|19039	10145	8.91	103	52	6	
B25	Lipid transfer protein 1	gi|19039	10145	8.91	493	52	6	
B26	Lipid transfer protein 1	gi|19039	10145	8.91	366	57	6	
C2	Exg1	gi|37926403	47335	4.45	254	20	7	
C3	Protein Z-type serpin	gi|1310677	43307	5.61	223	25	9	
C4	Protein Z-type serpin	gi|1310677	43307	5.61	278	20	8	
C5	Bgl2	gi|6321721	34118	4.16	154	6	1	R.NDLTASQLSDKINDVR.S
C6	Protein Z-type serpin	gi|1310677	43307	5.61	118	21	8	
C7	Protein Z-type serpin	gi|1310677	43307	5.61	154	25	11	
C8	Protein Z-type serpin	gi|1310677	43307	5.61	120	23	10	
C9	Trypsin/amylase inhibitor pUP13	gi|225102	15370	5.35	167	55	9	
C10	Trypsin/amylase inhibitor pUP13	gi|225102	15370	5.35	104	50	7	
C14	Trypsin inhibitor Cme precursor	gi|1405736	16341	7.49	99	29	5	
C15	Trypsin inhibitor Cme precursor	gi|1405736	16341	7.49	144	29	5	
C16	Alpha-amylase inhibitor BDAI-I	gi|123970	14045	5.36	211	38	7	
C17	Alpha-amylase inhibitor BDAI-I	gi|123970	14045	5.36	220	25	6	
C19	Alpha-amylase inhibitor BDAI-I	gi|123970	14045	5.36	182	25	5	
C22	Lipid transfer protein 1	gi|19039	10145	8.91	141	75	5	
C23	Lipid transfer protein 1	gi|19039	10145	8.91	223	40	3	
C24	Lipid transfer protein 1	gi|19039	10145	8.91	220	58	4	
C25	Lipid transfer protein 1	gi|19039	10145	8.91	241	68	4	
C26	Lipid transfer protein 1	gi|19039	10145	8.91	178	50	4	

^a^Protein identifications were confirmed with a significant MASCOT score of 71 for peptide mass fingerprint and ANOVA p ≤ 0.05, and a minimum of three matched peptides.

^b^Significant MS/MS score is > 54 for searches in *Saccharomyces cerevisiae*. Spectra’s for single peptide identifications are supplied in Additional file 1.

A general feature for all proteomes was that the proteins clustered in two regions on the gel, a region in the range of 36–42 kDa and one low molecular region from 8–20 kDa. Furthermore, a massively stained protein cluster at about pI 5.0-6.3 with a M_r_ of 37–42 kDa was identified in all gels. This protein cluster corresponded to the most abundant protein in beer - protein Z (Figure 3, Table 2). During fermentation of both beers, wort protein changes occurred. The protein spots identified as LTP1 (Figure 3; spot A22-A26, Table 2) on the wort 2-DE gel were more intense, than the corresponding spots on the 2-DE gel for the two beers. In the same pI range as LTP1 was detected, two lower molecular protein spots (Figure 3; spot A28, A29, Table 2) were detected in wort and identified as LTP2. These two LTP2 spots were undetectable in beer (Figure 3). Another feature that occurred during fermentation was that the serpin protein cluster of protein Z was shifted towards the acidic area, dividing the serpin protein cluster into two (Figure 3; B,C). This was not observed on the wort protein 2-DE gel (Figure 3; A).

Three protein spots found exclusively in beer were identified to be cell wall associated yeast proteins, Uth1 – involved in cell wall biogenesis (Figure 3; spot B1, Table 2, Additional file 1), Exg1 – an exo-β-1,3-glucanase, (Figure 3; spot B2, C2, Table 2) and Bgl2 - endo-β-1,3-glucanase (Figure 3; spot C5, Table 2, Additional file 1). In both beers, two higher molecular protein spots with a pI of 4.8 were observed and identified by MALDI-TOF-MS as Uth1 (55 kDa [Figure 3; spot B1, C1, Table 2]) and Exg1 (47 kDa [Figure 3; spot B2, C2, Table 2]). Although protein spots corresponding to Uth1 were observed in both beers, Uth1 was only identified in beer brewed with WLP001 (Figure 3; spot B1). In beer brewed with KVL011 a protein spot of 34 kDa (Figure 3; spot C5) was identified as Bgl2, which was not observed in the proteome of beer brewed with WLP001. However, Exg1 was identified in the beer brewed with both brewer’s yeast strains (Figure 3; spot B2, C2).

## Discussion

Several proteome analyses of beer [4,5,8,15,17], malt [8,14,22,23] and beer related processes [6,16] have been made, but none seem to have considered the influence of fermentation and brewer’s yeast strains on the beer proteome. To investigate if proteome changes from wort to beer were yeast strain dependent, proteins from wort and beer brewed with two different ale brewer’s yeast strains were separated by 2-DE and identified by MALDI-TOF-MS. It should be noted that the beers in this study are immature, that is beers that have not matured after fermentation. In the following, however, they will be referred to as beer.

The protein content of the beers were 0.29 mg/ml for KVL011 and 0.42 mg/ml for WLP001 (Table 1) placing them in the lower end of the range for a normal beer [24]. The concentration of wort proteins (0.50 mg/ml) is higher than for the brewed beers, indicating that proteins are either degraded proteolytically by the yeast during fermentation and/or precipitate with the yeast slurry.

The most recent proteome studies have identified 20–30 barley proteins in wort and beer [4-6]. In our study, nine unique proteins are identified out of 27 distinct protein spots analysed (Table 2). Many of the proteins have multiple spots, probably due to different protein modifications taking place during germination of barley grain, killing or wort boiling [11,25]. For example, protein Z appears as a dominant diffuse zone in a 2-DE gel probably due to glycosylation of lysine residues by Maillard reactions occurring under the roasting of malt [9,26]. All identified barley proteins are reported as protease resistant and heat stable, as most of them are protease inhibitors and have survived a more than one hour long hop boiling (Table 2) [7,8].

In the wort proteome, protein Z appears as a cluster of many spots, while in both beer proteomes this cluster is divided into two clusters (Figure 3). Division of the protein Z cluster into two in both beers indicates that yeast has an influence on the modifications of protein Z. This, however, remains to be further investigated.

LTP2 is present in two spots in the wort proteome (Figure 3; spot A28, A29) but absent in the two beer proteomes, although a faint spot is observed in beer brewed with KVL011 but not identified (Figure 3; spot C28). Many studies have shown that denatured and unfolded LTP1 in beer is degraded by yeast-derived proteinase A [27,28], which can explain why LTP2 disappears and a decrease in LTP1 intensity is observed in our study. Degradation of LTP1 is not a desired trait in beer production, as LTP1 is a key foam protein and in addition acts as an antioxidant in beer [29,30].

The three high molecular weight proteins, Uth1, Exg1 and Bgl2, found exclusively in beer after fermentation, are identified to be yeast proteins. Uth1 is involved in the cell wall biogenesis, oxidative stress response, and the protein resembles β-glucanases but no activity is reported [31,32]. Exg1 and Bgl2 are involved in the modification of the glucan network of the yeast cell wall [33]. It is reported that Exg1, Bgl2 and Uth1 are anchored to the yeast cell wall by di-sulphide bridges, as they are released from yeast cells upon treatment with reducing agents as DTT [34,35]. During wine fermentations, yeast cells release Exg1 and Bgl2 from the cell wall to the wine [36]. In beer, Fasilo *et al.* (2010) identified Exg1, Bgl2 and Uth1 among the 40 protein fragments, originating from *S. cerevisiae*[4], and very recently the presence of these three full-length proteins have also been identified in different commercial beers [5]. These data correspond well with our findings here.

In addition, we report for the first time that different brewer’s yeast strains render different beer proteomes; i.e. Exg1 and Bgl2 are identified in the KVL011 beers, whereas in the WLP001 beer only Exg1 is identified. These data strongly indicate that changes in the beer proteome are strain dependent.

Identification of released yeast di-sulphide anchored proteins Uth1, Exg1 and Bgl2 in beer indicates the existence of a reducing environment which can be beneficial for the beer quality by reducing and liberating cell wall anchored yeast proteins. Overexpression of β-glucanases, like Exg1 and Blg2, in genetically modified brewer’s yeast strains, have shown positive effects on filtration of beer, due to increased degradation of β-glucans interfering with filtration [37,38]. Also in wine fermentations, an elevated production of Exg1 has positive effects on the quality of the end product due to an increased production of volatile products [39]. Uth1 could be speculated to function as an antioxidant or chelator of transition metals in beer due to its conserved cysteine residue motive with a putative Fe-binding motive [31]. A controlled release of these cell wall anchored proteins could contribute to improved beer quality.

It should be stressed that our study, using immature beer, only reveals a very limited number of yeast proteins in the beer as compared to the reports of e.g. Fasoli *et al*. (2010) and Konecna *et al*. (2012). These authors investigate commercial beers that are most likely fully mature and pasteurized [4,5], although not specifically stated, thereby explaining the higher number of identified yeast proteins due to cell lysis.

## Conclusion

In this study we find that the proteome of immature beer is dependent on the brewer’s yeast strain used. These data suggest a potential of using different yeast strains to gain wanted protein-related traits of beer, such as e.g. filtration ability and oxidative stability.

## Competing interests

The authors declare that they have no competing interest.

## Authors’ contributions

TSB conducted all experiments and analyzed results. SJ contributed to analysis of proteome data and editing the manuscript. NA and TSB conceived the study and participated in its design, coordination, and draft of the manuscript. All authors have read and approved the manuscript.

## Supplementary Material

Additional file 1 MS/MS Spectra’s for single peptide identification. Click here for file
