# Supplementary material for: The impact of different ale brewer’s yeast strains on the proteome of immature beer
Source: BMC Microbiol. 2013 Sep 30;13:215. doi: 10.1186/1471-2180-13-215 (PMC3849757; doi:10.1186/1471-2180-13-215)
Supplement: Additional file 1 — MS/MS Spectra’s for single peptide identification. [file 1471-2180-13-215-S1.doc]

Spot B1: Uth1

|  | **Query** | **Observed** | **Mr(expt)** | **Mr(calc)** | **ppm** | **Miss** |  |  |  |  | **Peptide** |
| --- | --- | --- | --- | --- | --- | --- | --- | --- | --- | --- | --- |
|  | [1](http://www.matrixscience.com/cgi/peptide_view.pl?file=../data/20120120/FtoTIiaSt.dat&query=1&hit=1&index=gi|486485&px=1&section=5&ave_thresh=56&_ignoreionsscorebelow=0&report=20&_sigthreshold=0.05&_msresflags=1025&_msresflags2=2&percolate=-1&percolate_rt=0) | **1387.7209** | **1386.7136** | **1386.6164** | **70.1** | **0** |  |  |  |  | **K.TQWPSEQPSDGR.S** |

Spot C5: Bgl2

|  | **Query** | **Observed** | **Mr(expt)** | **Mr(calc)** | **ppm** | **Miss** |  |  |  |  | **Peptide** |
| --- | --- | --- | --- | --- | --- | --- | --- | --- | --- | --- | --- |
|  | [1](http://www.matrixscience.com/cgi/peptide_view.pl?file=../data/20120120/FtoTIiaTE.dat&query=1&hit=1&index=gi|6321721&px=1&section=5&ave_thresh=54&_ignoreionsscorebelow=0&report=20&_sigthreshold=0.05&_msresflags=1025&_msresflags2=2&percolate=-1&percolate_rt=0) | **1789.0260** | **1788.0187** | **1787.9013** | **65.7** | **1** |  |  |  |  | **R.NDLTASQLSDKINDVR.S** |
